# Effects of Luteinizing Hormone Releasing Hormone A2 on Gonad Development in Juvenile Amur Sturgeon, Acipenser schrenckii, Revealed by Transcriptome Profiling Analysis

**DOI:** 10.3389/fgene.2022.859965

**Published:** 2022-03-24

**Authors:** Weihua Lv, Shubo Jin, Dingchen Cao, Nianmin Wang, Xing Jin, Ying Zhang

**Affiliations:** Key Open Laboratory of Cold Water Fish Germplasm Resources and Breeding of Heilongjiang Province, Heilongjiang River Fisheries Research Institute, Chinese Academy of Fishery Sciences, Harbin, China

**Keywords:** *Acipenser* schrenckii, LH-A2, transcriptome, pituitary, gonad

## Abstract

*Acipenser schrenckii* is an economically important aquatic species whose gonads require particularly long times to reach sexual maturity. Luteinizing hormone plays important roles in gonad development, and luteinizing hormone releasing hormone A2 (LH-A2) is used as an oxytocin to promote ovulation in aquaculture of *A. schrenckii*. In this study, we aimed to determine the effects of LH-A2 on gonad development in juvenile *A. schrenckii* through transcriptome profiling analysis of the pituitary and gonads after LH-A2 treatment at a dose of 3 μg/kg. The 17β-estradiol (E2) levels gradually increased with LH-A2 treatment time, and significantly differed from those of the control group on days 5 and 7 (*p* < 0.01). However, the content of testosterone (Testo) gradually decreased with LH-A2 treatment time and showed significant differences on day 3 (*p* < 0.05), and on days 5 and 7 (*p* < 0.01), compared to those in the control group. Thus, LH-A2 promotes the secretion of E2 and inhibits the secretion of Testo. Transcriptome profiling analysis revealed a total of 2,883 and 8,476 differentially expressed genes (DEGs) in the pituitary and gonads, respectively, thus indicating that LH-A2 has more regulatory effects on the gonads than the pituitary in *A. schrenckii*. Signal transduction, global and overview maps, immune system, endocrine system and lipid metabolism were the main enriched metabolic pathways in both the pituitary and gonads. Sixteen important genes were selected from these metabolic pathways. Seven genes were co-DEGs enriched in both signal transduction and endocrine system metabolic pathways. The other co-DEGs were selected from the immune system and lipid metabolism metabolic pathways, and showed mRNA expression changes of >7.0. The expression of five DEGs throughout LH-A2 treatment was verified to show the same patterns of change as those observed with RNA-seq, indicating the accuracy of the RNA-seq in this study. Our findings provide valuable evidence of the regulation of gonad development of juvenile *A. schrenckii* by LH-A2 and may enable the establishment of artificial techniques to regulate gonad development in this species.

## Introduction

The Amur sturgeon (*Acipenser schrenckii*) is an important aquatic species providing substantial economic benefits in China. In 2019, the annual production of *A*. *schrenckii* reached 15,317 tons, accounting for approximately 15% of the total sturgeon aquaculture production (Zhang et al., 2020). The optimum aquaculture temperature of *A. schrenckii* is 18–22°C. The main distributed regions of *A. schrenckii* are in the Amur, Songhua and Heilongjiang Rivers (Li et al., 2012; Li et al., 2020), while the main aquaculture regions include Yunnan, Guizhou, Shandong and Hebei Provinces (Zhang et al., 2020). Sturgeon eggs are highly valuable and consequently are sometimes called “black gold.” Female sturgeons are therefore preferred in the sturgeon aquaculture industry. However, the long period required for *A. schrenckii* to reach sexual maturity poses a severe problem in the sustainable development of the artificial aquaculture industry for this species. The gonadal primordia of *A. schrenckii* are first observed at 60 days after hatching, and the gonads begin to differentiate at 170 days after hatching, on the basis of histological observations (Zhang et al., 2012). However, the time required for gonad maturity in *A. schrenckii* is 5–7 years for testis development and 9–12 years for ovary development under artificial aquaculture conditions (Qu et al., 2010). Therefore, the mechanisms of sex determination and reproduction of *A*. *schrenckii* must urgently be fully understood to establish artificial techniques to regulate ovarian development. Previous studies have identified many reproductive genes in *A*. *schrenckii*, and determined their potential functions in the sex-determination and reproduction mechanisms (Jin et al., 2016; Lv et al., 2021). However, more studies must be performed.

The hypothalamus–pituitary–gonad (HPG) axis in vertebrates regulates gonad maturity (Nagahama 2005; Kim et al., 2014). Gonadotropin-releasing hormone (GnRH) is secreted by the hypothalamus under normal conditions. GnRH promotes ovarian development through stimulating the secretion of follicle stimulating hormone and luteinizing hormone (LH) (Coccia and Rizzello 2008; Gründker and Emons 2021). LH is secreted by the anterior pituitary gonadotrophs and is classified as a gonadotropin promoting gonad development. LH binds specific transmembrane receptors localized primarily in the ovarian cells and subsequently promotes ovarian development. In the ovaries, LH is required to promote and mediate ovulation through regulating the synthesis of androgens in follicular theca cells. LH then helps maintain the secretion of progesterone after ovulation and is required for blastocyst implantation in the uterus (Paoli et al., 2020; Guo et al., 2021). LH is also used in infertility treatment in women (Soleimanifar et al., 2016; Orlova et al., 2017). LH was reported to be involved in the process of ovarian development in salmonid fish (Josep et al., 2000) and *Ictalurus punctatus* (Kristanto et al., 2009), while caused the sex reversal in *Monopterus albus* (Tang et al., 1974). The cDNA sequences of luteinizing hormone *β* were cloned from *Kryptolebias marmoratus* (Rhee et al., 2009), *Anguilla dieffenbachia* (Saito et al., 2003), and *Engraulis japonicus* (Ohkubo et al., 2010), and proven to be involved in the ovarian development. In sturgeon artificial aquaculture, luteinizing hormone releasing hormone A2 (LH-A2) is used as an oxytocin to promote ovulation in sturgeons. Previous studies have identified the essential regulatory roles of KiSS1 and gonadotropin-releasing hormone analogue (GnRH-a) in the regulation of the HPG axis, thus affecting ovarian development in *A*. *schrenckii* (Jin et al., 2016; Lv et al., 2021). However, the effects of LH-A2 in promoting ovarian development and ovulation remain unclear. The genes regulated by LH-A2 treatment must be determined. Understanding the effects of LH-A2 is essential for establishing artificial techniques to shorten ovarian development in *A*. *schrenckii*.

In this study, LH-A2 was injected into juvenile *A*. *schrenckii* at 60 days after hatching. The important metabolic pathways and genes regulated by treatment with LH-A2 at 3 μg/kg were identified through transcriptome profiling analysis of the gonads and pituitary. To assess the effects of LH-A2 on gonad development, we analysed the crucial differentially expressed genes (DEGs) throughout LH-A2 treatment by using quantitative real-time PCR (qPCR). The combined results provide valuable evidence for regulating gonad development in juvenile *A. schrenckii*.

## Materials and Methods

### Ethics Approval

All fish handling and experimental procedures involved in this study were approved by the Animal Care and Use Committee of the Heilongjiang River Fisheries Research Institute, Chinese Academy of Fishery Sciences, Harbin, on the basis of the relevant guidelines and regulations.

### Sample Collection

The *A*. *schrenckii* in this study were hatched from a full-sibling population, and fed at the Amur Sturgeon Breeding and Engineering Centre, Heilongjiang River Fisheries Research Institute, Chinese Academy of Fishery Sciences. The new hatching fishes were maintained in the aerated water at 16°C with a dissolved oxygen content of ≥6 mg/L, and were fed sturgeon commercial fodder purchased from Shandong Shengsuo Feed Technology Co., Ltd. Each day, fish were fed twice with 2% of their total weight. A total of 200 fishes with body weights of 41.29–44.19 g were collected from the full-sibling population at 60 days after hatching and randomly divided into two groups. The gonad differentiation and development sensitive period has been shown to begin 60 days after hatching (Zhang et al., 2012). LH-A2 was purchased from Ningbo Sansheng Pharmaceutical Co., Ltd. The control group was injected with the 0.9% physiological saline; the experimental group was injected with LH-A2 at a dose of 3 μg/kg, which is commonly used in our *A*. *schrenckii* aquaculture program to promote ovulation (Gao et al., 2020). LH-A2 was dissolved to 3 μg/μL in 0.9% physiological saline, then injected into the muscle through the first dorsal bone plate, according to the body weight of each fish. The amount of injected 0.9% physiological saline was also determined, on the basis of the body weight of each fish. Blood samples from 15 individuals of *A*. *schrenckii* were collected from the control group and LH-A2 group at 0, 1, 3, 5 and 7 days after injection for the measurement of testosterone (Testo) and 17β-estradiol (E2) content. A blood sample was drawn from the tail vein of each *A*. *schrenckii*. The blood samples from five individual *A*. *schrenckii* were pooled to form a biological replicate, and three replicates were examined. The pituitaries and gonads sample (*n* = 15) were collected from the control group and LH-A2 group at day 7 after injection, and transcriptome profiling analysis of the pituitary and gonads was performed between the control group and LH-A2 group. Five tissue samples were pooled to form one biological replicate, and three biological replicates were examined. Gonads and pituitaries of another 15 individuals were collected from the control group and LH-A2 group at 0, 1, 3, 5 and 7 days after injection, then subjected to qPCR analysis. Pituitary and gonads from five different *A*. *schrenckii* were pooled to form a biological replicate, and three biological replicates were analysed. The collected tissues were immediately frozen in liquid nitrogen until RNA extraction, to prevent RNA degradation.

### Measurement of Steroid Hormone

The pooled blood samples of *A*. *schrenckii* from days 1, 3, 5 and 7 after LH-A2 and 0.9% physiological saline injection were kept at 4°C for 4 h, and then centrifuged at 3,000 rpm/min for 5 min to extract serum. E2 and Testo were then extracted from the serum with 5 ml 100% methyl alcohol. The content of E2 and Testo was measured with a BECKMAN ACESS II T Kit on a Beckman Coulter Access two instrument (Kraemer Boulevard Brea, CA, United States), according to the manufacturer’s protocol (Jin et al., 2019; Lv et al., 2021). All samples were run in triplicate.

### Transcriptome Profiling Analysis

The DEGs regulated by LH-A2 treatment were identified through transcriptome profiling analysis of the pituitary and gonads in *A*. *schrenckii*. The Illumina High-seq 2500 sequencing platform, which is widely used in transcriptome studies, was used to perform transcriptome profiling analysis. Previous studies have described the detailed procedures for RNA-seq and analysis (Jin et al., 2013; Jin et al., 2021). The Trinity program (version: trinityrnaseq_r20131110) was used to assemble the clean data into non-redundant transcripts (Grabherr et al., 2011). Gene annotation was then performed in the non-redundant (Nr) database, and the Gene Ontology (GO) (Ashburner et al., 2000), Cluster of Orthologous Groups (COG) (Tatusov et al., 2003) and Kyoto Encyclopaedia of Genes and Genomes (KEGG) databases (Minoru et al., 2008), with an E-value of 10^–5^ (Jin et al., 2013). The EB-seq algorithm was used to filter the differentially expressed genes, according to the criterion of false discovery rate <0.05 (Benjamini et al., 2001). The transcriptome raw reads were annotated in the *Acipenser ruthenus* genome by using Cufflinks (Trapnell et al., 2010).

### qPCR Analysis

qPCR analysis was used to verify the reliability of the RNA-seq data of selected DEGs, regulated by LH-A2. Previously published studies have described the detailed procedures of qPCR analysis (Zhang et al., 2013; Jin et al., 2019). Briefly, total RNA was extracted from each tissue, using the UNlQ-10 Column Trizol Total RNA Isolation Kit (Sangon, Shanghai, China) following the manufacturer’s protocol. A total of 1 μg total RNA from each tissue was used to synthesize the cDNA template by using the PrimeScript™ RT reagent Kit (Takara Bio Inc., Japan). The expression level of each tissue was determined using the UltraSYBR Mixture (CWBIO, Beijing, China). The qPCR analysis was performed on a Bio-Rad iCycler iQ5 Real-Time PCR System (Bio-Rad), and SYBR Green RT-qPCR assays were used. The primers for qPCR analysis are listed in Table 1 *β*-actin was used as the reference gene in this study (Shi et al., 2010; Wu et al., 2020). The qPCR reaction was 95°C for 10 min, followed by 40 cycles of 95°C for 15 s and 60°C for 1 min. DEPC-water was used as a negative control instead of the template. The relative expression levels were measured with the 2^−ΔΔCT^ method (Livak and Schmittgen 2001).

**TABLE 1:** Primers used in this study.

Gene	Sequence	Melt temperature (°C)	Efficiency (%)
PKC	F: GGA​GAA​CAT​CAT​CCT​GGC​CA	60	97.9
	R: TCC​TTG​AGG​CTG​TCG​TTG​TG		
Src	F: AGT​ACC​ACA​GCA​AGG​TCA​GC	60	98.2
	R: AGA​ACC​AAT​GTC​GCT​CTG​GG		
Trx	F: AAC​AAG​ATC​AAG​ACG​GGC​GA	60	99.1
	R: AAC​CGC​TCC​ATG​TCG​ATC​AA		
Claudin 4	F: TGT​GAC​AGT​GGC​TGT​ACG​TT	60	98.8
	R: AAC​CGC​CTG​GAT​GAT​GAA​CA		
ADH3	F: ATG​AAT​CAC​TAC​TGG​CGC​GA	60	98.5
	R: CAG​GTT​GTC​TTG​GAA​ACG​CA		
β-actin	F: ATC​GCC​GCA​CTG​GTT​GTT​GA	60	97.6
	R: ATG​CCG​TGC​TCG​ATG​GGA​TA		

### Statistical Analysis

All statistics were measured in SPSS Statistics 23.0. Quantitative data are expressed as the mean ± SD. Statistical differences were estimated by one-way ANOVA followed by LSD and Duncan’s multiple range test for qPCR analysis in different mature tissues. The statistical significance of differences in Testo and E2 on the same day between the control group and experimental group, and verification of RNA-seq data were determined with paired t-tests. A probability level of 0.05 was considered to indicate significance (*p* < 0.05).

## Results

### Measurement of Steroid Hormone

**FIGURE 1:**
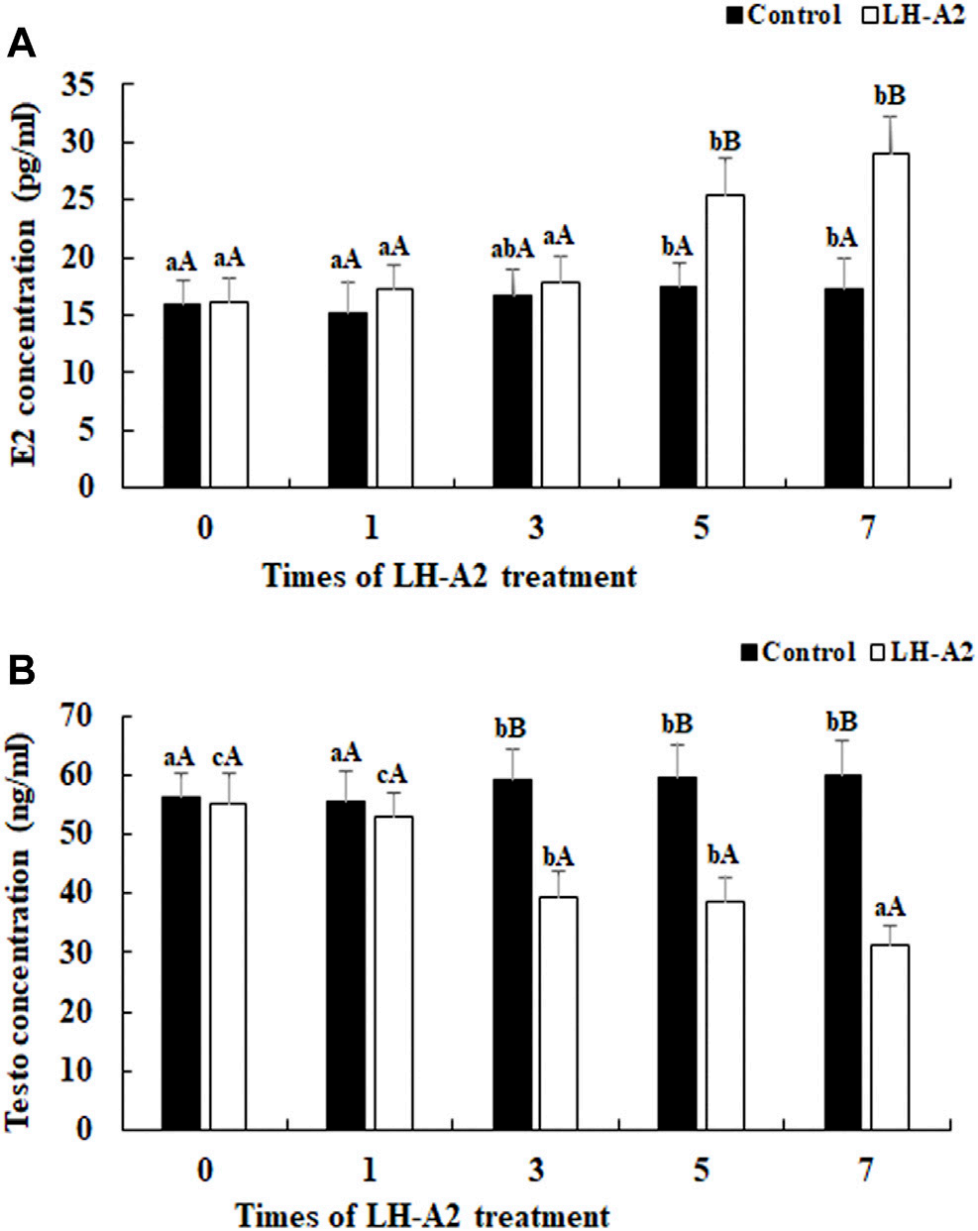
The content of E2 and Testo level at different time points after the treatment of LH-A2 at the dose of 3 μg/kg. Lowercases indicated the signifcant difference between different time points in the same treated group, and capital letters indicated the significant difference between control group and LH-A2 group on the same day (*p* < 0.05). **(A)** The content of E2; **(B)** The content of Testo.

The effects of LH-A2 on the secretion of E2 and Testo in the serum in juvenile *A*. *schrenckii* are shown in Figure 1. The content of E2 gradually increased with LH-A2 treatment time at a dose of 3 μg/kg. The content of E2 reached a peak at 7 days after LH-A2 treatment, and was different from that at the other tested time points except 5 days (*p* < 0.05). The contents of E2 at days 5 and 7 after LH-A2 treatment showed significant difference with those of the control group on the same day (*p* < 0.01) (Figures 1A). However, the changes in Testo showed an opposite secretion pattern from that of E2, revealing a gradual decrease with LH-A2 treatment time. The highest content of Testo was observed at 0 days. The content of Testo was different from that in the control group at 3, 5 and 7 days after LH-A2 treatment (*p* < 0.05) (Figures 1B).

### Length Distribution

Illumina Hiseq2500 was used to produce reads for clustering and *de novo* assembly. A total of 66.5 Gb raw reads were generated. Approximately 63.4 Gb clean reads remained after elimination of adapter sequences and filtering out low-quality reads (the number of bases in each read was less than 25 bp). The De novo program was used to assemble the *A. schrenckii* transcriptome. A total of 140,769 unigenes were assembled with a mean length of 967 bp. Most unigenes (34.2%) were 300–400 bp in length, followed by 400–500 bp (12.6%) and >3,000 bp (8.8%).

### Functional Annotation

All assembled unigenes were compared with the non-redundant protein database and nucleotide sequences in NCBI to identify their putative functions, by using Blastp and Blastx at an E-value of <10^–5^. A total of 140,769 unigenes were assembled in this study, 54,590 of which were annotated in the Nr database. Approximate 70% of the raw reads were highly matched with the *A. ruthenus* genome. A total of 13,736 unigenes were finally annotated in the *A. ruthenus* genome. The other unannotated unigenes maybe caused by the analysis without reference genome whose functions have not yet been identified. The functions of these unannotated transcripts require further investigation.

**FIGURE 2:**
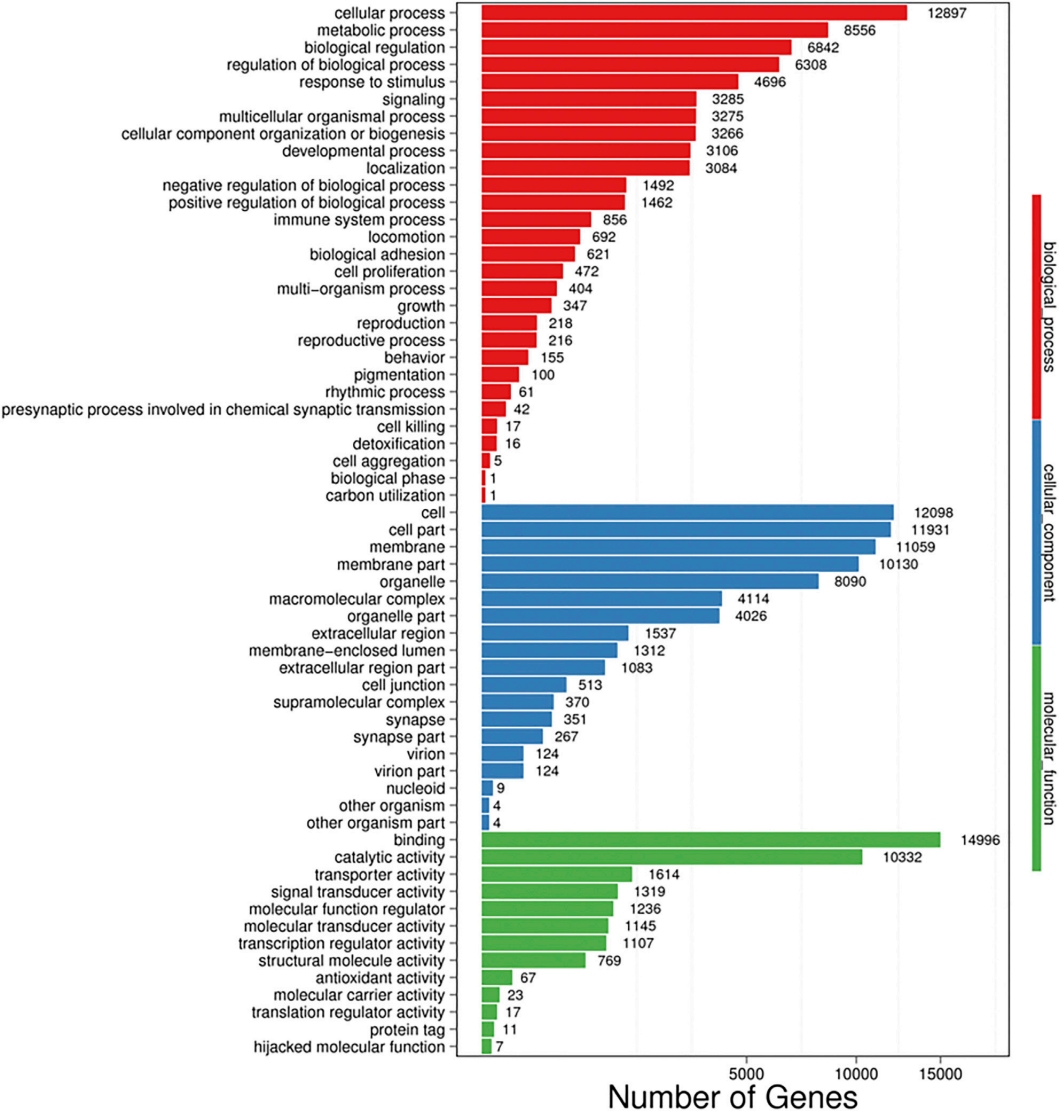
Gene ontology (GO) analysis of all unigenes identified by the transcriptome analysis. The left *y*-axis indicates the percentage of a specific category of proteins existed in the main category, whereas the right *y*-axis indicates the number of a specific category of proteins existed in main category. The matched unigenes were divided into three categories, including biological process (62,493), cellular component (67,146), and molecular function (32,643). The matched unigenes were comprised of 61 functional groups, in which the number of unigenes in each functional group ranged from 1 to 14,996. Binding; Cellular process, Cell, Cell part and membrane represent the top five functional groups.

**FIGURE 3:**
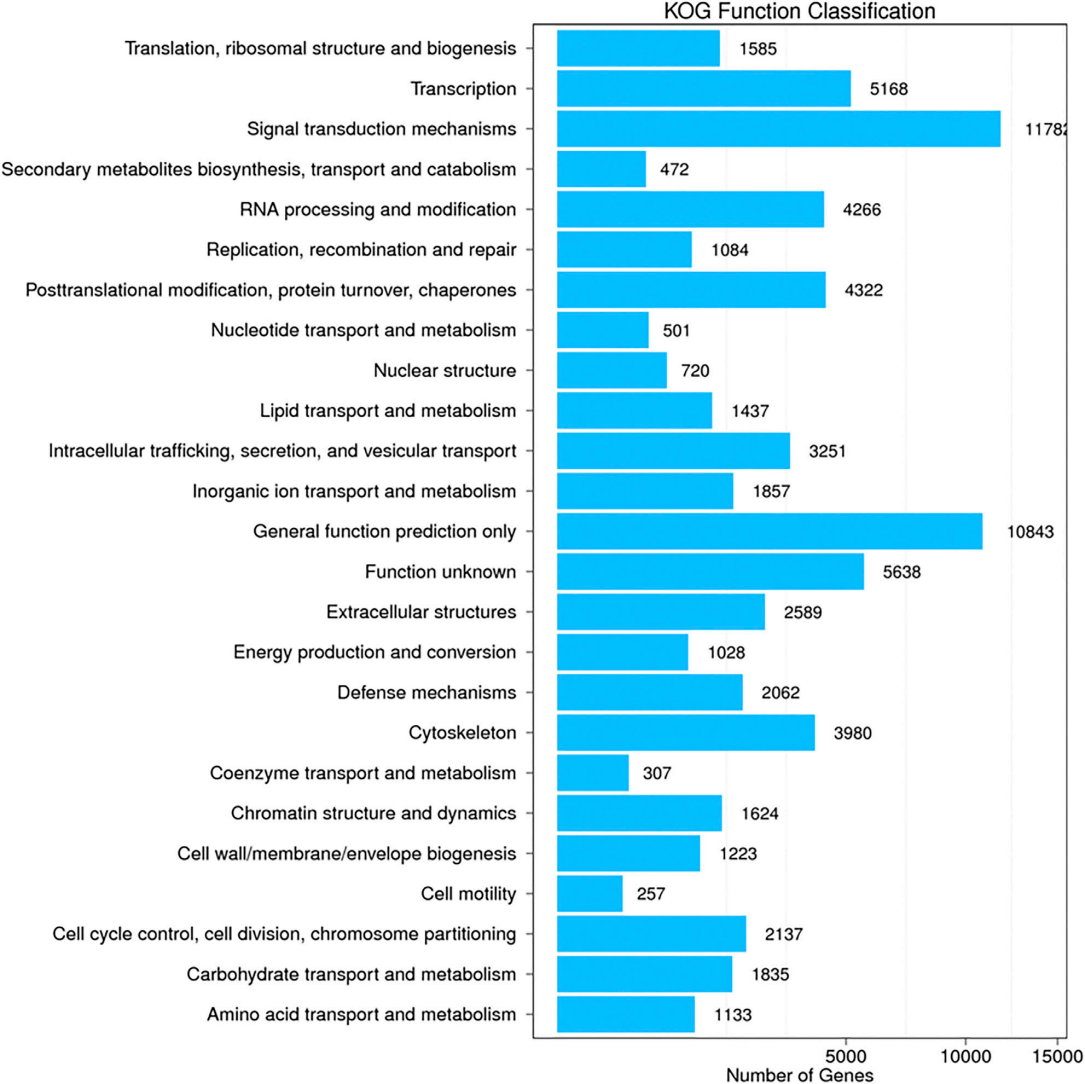
Cluster of orthologous groups (COG) classification of all unigenes identified by the transcriptome analysis. The matched unigenes were classified functionally into 25 functional categories in the COG database. The number of unigenes in each functional category ranged from 257 to 11782.

Additional functional analysis of these unigenes was performed with the GO, COG and KEGG pathway databases. GO and COG provide a structured, controlled vocabulary for describing the functions of gene products. A total of 29,622 unigenes (Figure 2) and 44,515 unigenes (Figure 3) matched known proteins in the GO database and COG database, respectively. A total of 50,177 unigenes matched known proteins in the KEGG database and were divided into 258 metabolic pathways.

### Identification of Differentially Expressed Genes

Transcriptome profiling analysis of the gonads and pituitary was performed between 0.9% physiological saline treated *A. schrenckii* and LH-A2 treated *A. schrenckii*, to select the genes and metabolic pathways involved in gonad development in juvenile *A. schrenckii*. A total of 2,883 genes were differentially expressed in the pituitary, including 1,612 upregulated genes and 1,271 downregulated genes in LH-A2 treated *A. schrenckii* (criteria of >2.0 for upregulation and <0.50 for downregulation, and *p*-value < 0.01) (Supplementary Table S1). The DEGs were then blasted against the GO and KEGG database. A total of 1,005 DEGs were assigned to the GO database, comprising 49 functional groups. Binding, cellular process, cell, cell part and membrane represented the main functional groups, in which the number of DEGs exceeded 350. A total of 1,009 DEGs matched the known proteins in the KEGG database and were divided into 44 metabolic pathways. Signal transduction, global and overview maps, infectious diseases: viral, immune system and endocrine system were the most enriched metabolic pathways in the gonad.

A total of 8,476 DEGs were identified in the gonads, including 3,748 that were upregulated and 4,728 that were downregulated in LH-A2 treated *A. schrenckii*, according to the same criteria as those for the pituitary (Supplementary Table S2). Among these 8,476 DEGs, 2,417 were assigned to the GO database. These DEGs comprised 55 functional groups, and the number of DEGs in each functional group ranged from 1 to 1,115. Binding, cellular process, catalytic activity, membrane, cell and cell part were the main functional groups, in which the number of DEGs exceeded 850. A total of 2,540 DEGs matched the known protein in KEGG database and were divided into 43 metabolic pathways. Signal transduction, global and overview maps, immune system, endocrine system and lipid metabolism were the main enriched metabolic pathways in the pituitary.

### Identification of Genes for Gonad Development

The strong candidate genes potentially involved in gonad development of *A*. *schrenckii* are listed in Table 2. These genes were selected from the main enriched metabolic pathways of DEGs in both pituitary and gonad. The co-DEGs were identified as the genes differentially expressed in both the pituitary and gonads. Seven co-DEGs were enriched in both signal transduction and endocrine system metabolic pathways, four of which were upregulated and three of which were downregulated after LH-A2 treatment. The other co-DEGs were identified from the immune system and lipid metabolism metabolic pathways, and the upregulated expression changes were >7.0.

**TABLE 2:** Selected DEGs involved in the gonad development of *A*. *schrenckii*.

Gene	*p*-value	Accession number	Metabolic pathway	Folder change (LH-A2 vs Control)	
				Pituitary	Gonad
Proto-oncogene tyrosine-protein kinase Src	3.65E-12	XP_014023654.1	Signal transduction; Endocrine system	7.16	6.32
Protein kinase C	1.74E-05	XP_006633404.1	Signal transduction; Endocrine system	4.31	6.32
Nuclear receptor subfamily 4	2.47E-42	XP_018616202.1	Signal transduction; Endocrine system	2.36	3.52
HRAS-like suppressor 3	6.43E-202	XP_015218238.1	Signal transduction; Endocrine system	3.69	2.79
Collagen alpha-1	3.57E-05	EMP35428.1	Signal transduction; Endocrine system	−2.36	−2.11
Transcription factor 7	4.56E-30	XP_006630833.1	Signal transduction; Endocrine system	−2.15	−2.83
Ficolin-1	1.71E-05	EMP28399.1	Signal transduction; Endocrine system	−2.13	−2.79
Alcohol dehydrogenase class-3	5.51E-25	XP_006629873.1	Lipid metabolism	8.12	10.15
Prostaglandin f synthase	2.06E-08	ORC86208.1	Lipid metabolism	7.08	7.56
Beta domain protein	2.46E-05	KHJ90253.1	Lipid metabolism	7.04	8.51
Pol protein	1.55E-05	AAC16764.1	Lipid metabolism	8.37	10.82
Thioredoxin	4.10E-07	XP_012248332.1	Immune system	9.34	8.21
Foldase protein	2.46E-05	XP_017036539.1	Immune system	11.37	7.21
Microtubule-associated protein	1.48E-16	XP_016837448.1	Immune system	7.21	7.89
DNA-directed RNA polymerase	4.39E-29	CDW59665.1	Immune system	11.36	8.69
Claudin-4	5.46E-12	XP_006640934.1	Immune system	8.31	11.42

### qPCR Analysis

**FIGURE 4:**
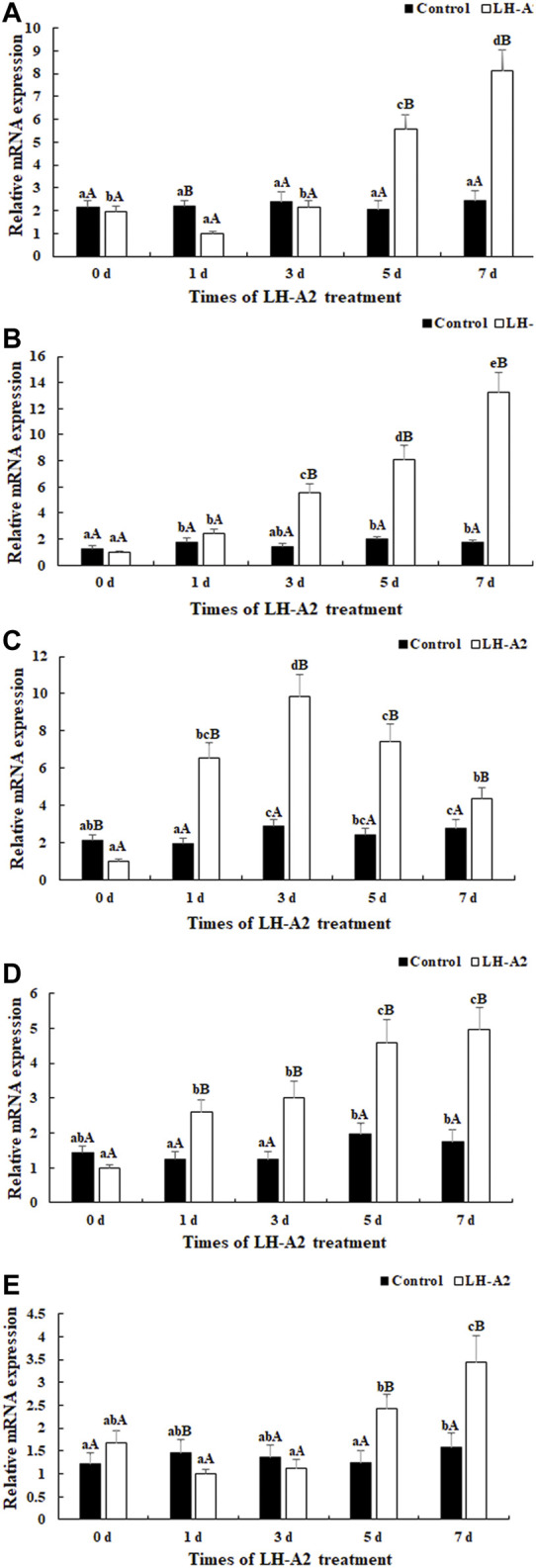
Expression characterization of five DEGs in pituitary at different time points after the LH-A2 treatment. The amount of five DEGs mRNA was normalized to the *β*-actin transcript level. Data are shown as mean ± SD (standard *deviation*) of tissues from three biological replicates. Lowercases indicated the signifcant difference between different time points in the same treated group, and capital letters indicated the significant difference between control group and LH-A2 group on the same day (*p* < 0.05). **(A)** Expression characterization of As-PKC; **(B)** Expression characterization of *As-Src*; **(C)** Expression characterization of As-Trx; **(D)** Expression characterization of As-Claudin4; **(E)** Expression characterization of As-ADH3.

Five DEGs were selected for qPCR analysis throughout LH-A2 treatment: Protein kinase C (PKC), Proto-oncogene tyrosine-protein kinase Src (Src), Thioredoxin (Trx), Claudin-4 and Alcohol dehydrogenase class-3 (ADH-3). The expression of these five tested DEGs generally remained stable at various time points after the treatment with 0.9% saline in the pituitary, as compared with the changes under LH-A2 treatment. The expression of *As-Src* and As-Claudin4 gradually increased with LH-A2 treatment time. The expression of As-PKC and As-ADH3 slightly decreased 1 day after LH-A2 treatment, then significantly increased and reached a peak after 7 days of LH-A2 treatment. However, the expression of As-Trx significantly increased from 0 to 3 days after LH-A2 treatment, and then gradually decreased by 7 days. The expressions of these five tested DEGs at day 5 and day 7 were significantly lower in the 0.9% saline group than the LH-A2 group (*p* < 0.05), in agreement with the RNA-seq results (Figure 4).

**FIGURE 5:**
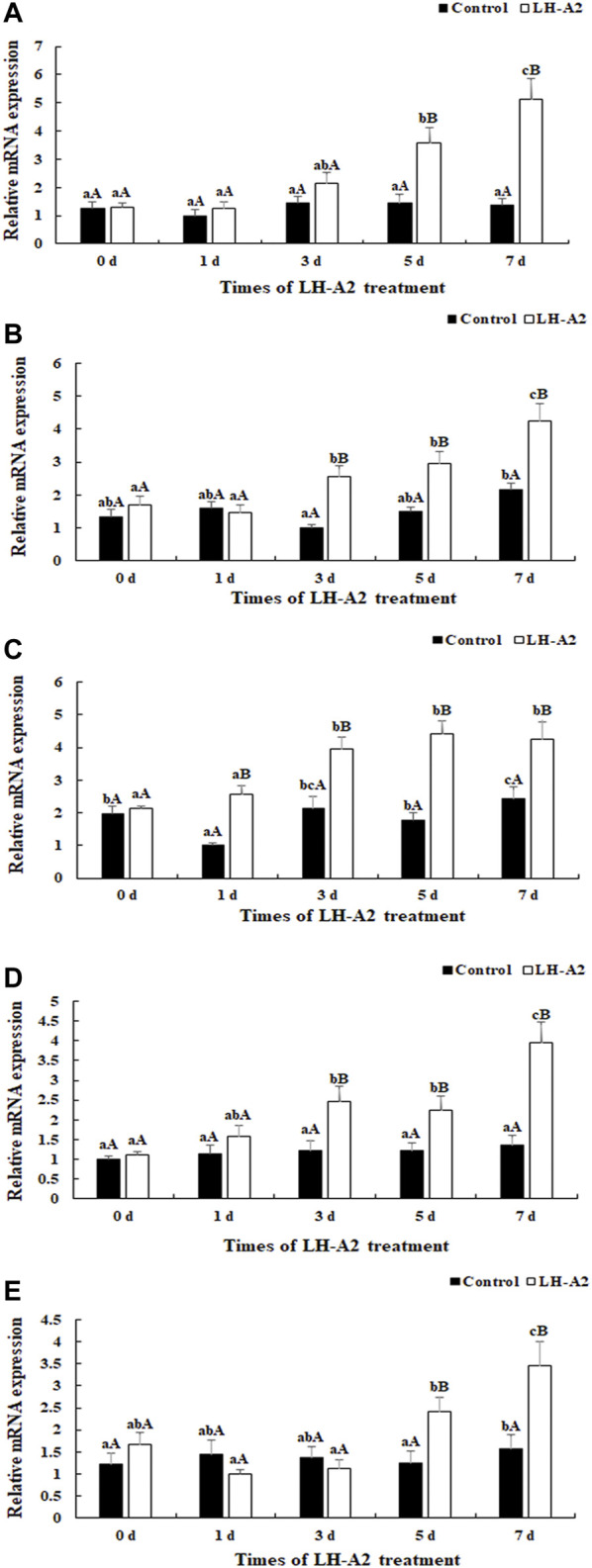
Expression characterization of five DEGs in gonad at different time points after the LH-A2 treatment. The amount of five DEGs mRNA was normalized to the *β*-actin transcript level. Data are shown as mean ± SD (standard *deviation*) of tissues from three biological replicates. Lowercases indicated the signifcant difference between different time points in the same treated group, and capital letters indicated the significant difference between control group and LH-A2 group on the same day (*p* < 0.05). **(A)** Expression characterization of As-PKC; **(B)** Expression characterization of *As-Src*; **(C)** Expression characterization of As-Trx; **(D)** Expression characterization of As-Claudin4; **(E)** Expression characterization of As-ADH3.

In gonads, the expression of As-PKC, *As-Src*, As-Trx and As-Claudin4 gradually increased with LH-A2 treatment time, whereas the expression of As-ADH3 slightly decreased at 1 day after LH-A2 treatment, then significantly increased and reached a peak at 7 days after LH-A2 treatment. The expression changes in these five DEGs in the gonads were similar to those in the pituitary, showing significantly lower expression in the 0.9% saline group than the LH-A2 group at days 5 and 7 (*p* < 0.05), in agreement with the RNA-seq results (Figure 5).

## Discussion

In the present study, through transcriptome profiling analysis, we aimed to select the important genes and metabolic pathways in the gonads and pituitary regulated by LH-A2, which is widely used in sturgeon aquaculture to promote ovulation. LH has been proven to be involved in the process of gonad development and ovulation in many aquaculture species (Tang et al., 1974; Josep et al., 2000; Kristanto et al., 2009). Our previous study identified the important regulatory roles of KiSS1 in the HPG axis (Jin et al., 2016; Lv et al., 2021). However, the regulatory roles of LH-A2 in *A. schrenckii* remained unclear. The biological functions of genes regulated by LH-A2 treatment must be further investigated, especially those of the up-regulated genes. There genes may play essential roles in the ovarian maturity and development of *A*. *schrenckii* and thus may support the development of artificial techniques to regulate gonad development in this species.

In the present study, the content of E2 increased with LH-A2 treatment time at a dose of 3 μg/kg, whereas the content of Testo decreased. LH is required for ovarian maturity and ovulation in vertebrates. LH-A2 is widely used as an oxytocin to promote ovulation in sturgeon aquaculture programs. E2, which is produced and secreted by the granulosa cells of the ovarian follicles, promotes female differentiation and sexual development (Hodgin et al., 2001; Maggiolini et al., 2004). Testosterone is a major sex differentiation hormone in vertebrates, and is commonly detected in the haemolymph and testis. Testosterone is essential for sexual development in males (Shalender and Stuart 2019; Huang et al., 2021). The dose of LH-A2 at 3 μg/kg stimulated the secretion of E2 in *A*. *schrenckii* and inhibited the secretion of Testo, thus indicating that LH-A2 is involved in ovarian maturity in *A*. *schrenckii*.

A total of 140,769 unigenes were assembled, substantially more than in previous studies; therefore, this study provides valuable information for the analysis of gonad development in *A. schrenckii* (Jin et al., 2016; Lv et al., 2021). Approximate 70% raw reads were highly matched with the *Acipenser ruthenus* genome, and a total of 13,736 unigenes were finally annotated in the *A. ruthenus* genome, indicating *A. schrenckii* has close evolutionary relationship with *A. ruthenus* (Cheng et al., 2019). A total of 2,883 DEGs and 8,476 DEGs were identified in the pituitary and gonads, respectively, after the injection of LH-A2. Therefore, LH-A2 has more regulatory roles in the gonads than the pituitary. GO analysis of DEGs revealed that binding, cellular process, catalytic activity, membrane, cell and cell part were the main functional groups regulated by LH-A2, according to transcriptome profiling analysis of both the pituitary and gonads. Consequently, the genes involved in gonad development in *A. schrenckii* were mainly enriched in these functional groups.

KEGG analysis of DEGs revealed a total of 187 and 74 DEGs in the gonads and pituitary, respectively, which were involved in the most enriched metabolic pathways in the transcriptome profiling analysis of the pituitary and gonads. Thus, signal transduction and endocrine system metabolic pathways may play essential roles in gonad development in juvenile *A*. *schrenckii*, as well as the DEGs in these metabolic pathways. The endocrine system includes various endocrine glands, including the hypothalamus, pituitary, pineal, thyroid, parathyroid, adrenal, pancreas, ovaries and testes. These glands can secrete nitrogen-containing hormones and steroid hormones (Garcia-Reyero 2018; Yuan et al., 2021). The bloodstream carries hormones from the organs where they are produced to the organs that they affect. Each hormone influences an organ or a type of cells within an organ, which is known as the target organ or target cell (Garcia-Reyero 2018). Signal transduction involves numerous elements, all playing essential roles in target cells in the recognition of their specific hormones (McIlwraith and Belsham 2020). Organs transfer chemical signals through blood-borne transmission. The target cells have receptors that specifically bind the corresponding hormones and exert effects after hormones binding. A reasonable explanation for this is that the metabolic pathways work together to recognise the specific hormone, in order to promote the ovarian development and ovulation in *A*. *schrenckii*. We selected seven co-DEGs enriched in the signal transduction and endocrine system metabolic pathways in both the gonads and pituitary. Among these DEGs, four were upregulated in the gonads and pituitary, whereas the other three were downregulated. The expressions of PKC and Src were up-regulated after LH-A2 treatment, which may affect the ovarian development and ovulation in *A*. *schrenckii*. PKC activation plays an important role in controlling the functions of other proteins in multiple signal transduction cascades. PKC was initially defined as a participant in the regulation of hyperglycaemia (Gopalakrishna and Jaken 2000; Inoguchi et al., 2000). PKC activation was further identified to regulate several biological processes, including the inhibition of eNOS expression in endothelial cells (Kuboki et al., 2000), the stimulation of VEGF expression in vascular smooth muscle cells (Williams et al., 1997), a decrease in NO production in smooth muscle cells (Ganz and Seftel 2000) and the activation of NF-κB (Ha et al., 2002). Src has been identified to be an important factor with a wide range of biological functions, including cell proliferation, adhesion, angiogenesis, organisation of the cell skeleton, cell division and cell death (Dunant and Ballmer-Hofer 1997; Parsons and Parsons 2004; Ingley 2008; Tegtmeyer and Backert 2011; Kinsey 2014). Src has also been reported to participate in the treatment of ILT herpesvirus (Li et al., 2016) and macrophage-myofibroblast transition-driven fibrotic diseases (Tang et al., 2018).

The immune system and lipid metabolism were two major metabolic pathways enriched in both the pituitary and gonads. The immune system involves complex mechanisms of defence responses, and is found in humans and other advanced vertebrates, in which it promotes stress resistance. The nonspecific defence system (innate immunity) and specific defence system (acquired immunity) work together in organisms to prevent microorganisms from entering and proliferating within the body. Nonspecific protective mechanisms target all microorganisms equally, whereas specific immune responses are tailored to particular types of invaders. These immune mechanisms also help eliminate abnormal cells in the body (Ader et al., 1995; Estrada et al., 2021). A reasonable explanation for the enrichment of the immune system is that LH-A2 treatment might be involved in gonad development. Thus, aged or abnormal cells must be digested, to adapt the LH-A2 treatment. A total of 24 co-DEGs were selected, five of which were upregulated in the LH-A2 treated group, with an expression change >7.0. Tight junctions, the main apical component of intercellular junctional complexes, play essential roles in establishing cell polarity and paracellular permeability (Tsukita and Furuse 2000). The Claudin family forms integral constituents of tight junctions (Morita et al., 1999), consisting of at least 20 transmembrane proteins, and are a major factor in establishing the intercellular barrier (Heiskala et al., 2001). Claudin-4 is an integral constituent of tight junctions and is overexpressed in pancreatic cancer (Michl et al., 2001; Michl et al., 2003) and ovarian cancer (Rangel et al., 2003). Overexpression of Claudin-4 has also been reported to regulate the levels of Claudin-1, -2, or -3, occludin or ZO-1 (Itallie et al., 2001). Trx is a ubiquitous disulfide reductase responsible for maintaining proteins in their reduced state (Holmgren 1985). Trx is a negative regulator of Apoptosis signal-regulating kinase 1 (Saitoh et al., 1998). In addition, thioredoxin-interacting protein is associated with oxidative stress and participates in the pathogenesis of type 2 diabetes (Zhou et al., 2010).

Lipid metabolism is another important metabolic pathway enriched in both the pituitary and gonads. Lipid metabolism is a complicated process regulating lipid synthesis and degradation. It is controlled by many bioregulators from the pituitary, liver, endocrine pancreas, adipose tissue and the gut microbiome. Lipid metabolism is regulated by several hormones, as well as the presence of cancer or pregnancy. Leptin affects lipid metabolism through regulating the mRNA levels and concentrations of enzymes such as acetyl-CoA carboxylase in adipocytes (Dahl et al., 2019; Markevich et al., 2021). A reasonable explanation for this is that lipid metabolism provided energy for ovarian development after the treatment of *LH-A2* in *A*. *schrenckii*. A total of co-13 DEGs were selected, four of which were upregulated in the LH-A2 treated group, with an expression change >7.0. Alcohol dehydrogenase (ADH) is a principal enzyme participating in the oxidation of ingested ethanol in humans (Cañestro et al., 2010). ADH3 has been found to be involved in the synthesis of retinoic acid in chordates (Dong et al., 1996).

Five DEGs from these main enriched metabolic pathways were selected for qPCR verification throughout LH-A2 treatment. This study reports the first analysis of the expression of these five genes under regulation by LH-A2 treatment. qPCR analysis revealed that the expression of *As-Src* and As-Claudin4 gradually increased with LH-A2 treatment time in the pituitary, whereas As-PKC and As-ADH3 required several days to respond to the regulation by LH-A2. Interestingly, LH-A2 upregulated the expression of As-Trx for only several days. In gonads, only As-ADH3 required several days to respond to regulation by LH-A2, whereas the expression of the other four DEGs gradually increased with LH-A2 treatment time. This finding also indicated that LH-A2 has more essential regulatory roles in the gonads than the pituitary in *A. schrenckii*.

In conclusion, the measurements of the content of E2 and Testo after LH-A2 treatment revealed that LH-A2 stimulates the secretion of E2 while inhibiting the secretion of Testo in *A. schrenckii*. These results are consistent with findings from aquaculture indicating that LH-A2 promotes ovulation in *A. schrenckii*. Transcriptome profiling analysis revealed a total of 2,883 and 8,476 in the pituitary and gonads, respectively, indicating that LH-A2 has more regulatory effects on the gonads than the pituitary. Transcriptome profiling analysis also revealed that the metabolic pathways of signal transduction, global and overview maps, immune system, endocrine system and lipid metabolism, and their enriched upregulated co-DEGs, may play essential roles in ovarian development in *A. schrenckii*. qPCR analysis revealed that LH-A2 stimulated the expression of these tested co-DEGs at 7 days after treatment in the pituitary and gonads, findings consistent with those of RNA-seq, whereas differences were observed in the regulatory processes. The genes, which were rapidly responded to the LH-A2 treatment, may play essential regulatory roles in gonad development in *A. schrenckii*. The biological functions of these genes need further investigation in *A. schrenckii*. The artificial technique to regulate the process of ovarian development maybe established in *A. schrenckii* through affecting the expressions of these selected genes. This study identified the effects of LH-A2 in *A. schrenckii*, thus providing valuable evidence for establishing artificial techniques to regulate gonad development in *A. schrenckii*.

## Data Availability

The datasets presented in this study can be found in online repositories. The names of the repository/repositories and accession number(s) can be found in the article/Supplementary Material.
